# Molecular Basis for the Therapeutic Effects of Exercise on Mitochondrial Defects

**DOI:** 10.3389/fphys.2020.615038

**Published:** 2021-01-13

**Authors:** Jonathan M. Memme, David A. Hood

**Affiliations:** ^1^Muscle Health Research Centre, York University, Toronto, ON, Canada; ^2^School of Kinesiology and Health Science, York University, Toronto, ON, Canada

**Keywords:** mitochondrial quality control, skeletal muscle, aging, cancer, exercise as medicine

## Abstract

Mitochondrial dysfunction is common to many organ system disorders, including skeletal muscle. Aging muscle and diseases of muscle are often accompanied by defective mitochondrial ATP production. This manuscript will focus on the pre-clinical evidence supporting the use of regular exercise to improve defective mitochondrial metabolism and function in skeletal muscle, through the stimulation of mitochondrial turnover. Examples from aging muscle, muscle-specific mutations and cancer cachexia will be discussed. We will also examine the effects of exercise on the important mitochondrial regulators PGC-1α, and Parkin, and summarize the effects of exercise to reverse mitochondrial dysfunction (e.g., ROS production, apoptotic susceptibility, cardiolipin synthesis) in muscle pathology. This paper will illustrate the breadth and benefits of exercise to serve as “mitochondrial medicine” with age and disease.

## Introduction

Mitochondria are the principal organelles tasked with the provision of cellular energy in the form of ATP, which is supplied in equilibrium with the energy demands placed on the cell. Changes within the cellular environment also provoke the mitochondrial network to regulate other important functions, such as initiating mitochondrial-nuclear retrograde signals, maintaining redox and Ca^2+^ homeostasis, as well as determining cell fate (Hood et al., 2019). The complex and multifaceted influence of mitochondria on cellular functions requires that the organelle network exist in a constant state of flux that allows for precise remodeling in order to match morphology and function of mitochondria to these imposing demands (Eisner et al., 2018; Guan et al., 2019). An intriguing aspect of mitochondrial biology is the tissue specificity that these organelles exhibit (Fernández-Vizarra et al., 2011). Skeletal muscle in particular comprises a large volume of total body mass (∼40%) and is highly metabolic with varying concentrations of mitochondria that provide ATP to the working muscle for locomotion, posture, and the generation of fine motor skills. Additionally, skeletal muscle displays distinct mitochondrial characteristics that are dependent on the location within the cell, and the metabolic requirements within those cellular compartments. Thus, given the relative proportion of body mass and the metabolic nature of skeletal muscle, the status of the mitochondrial pool within this tissue has considerable ramifications for systemic health.

Within the skeletal muscle myofiber, mitochondria exist in two distinct locales, which correspond with unique conformations and roles (Kayar et al., 1988; Cogswell et al., 1993; Ferreira et al., 2010). Subsarcolemmal (SS) mitochondria appear to be essential to provide energy for nuclear gene transcription and transport across the membrane (Kirkwood et al., 1986; Ogata and Yamasaki, 1997). Intermyofibrillar (IMF) mitochondria, localized along the sarcomeric proteins of the myofibrils and exhibit a more elongated appearance forming a reticular network throughout muscle fibers in close proximity to the transverse tubules and the sarcoplasmic reticulum (Kirkwood et al., 1986; Ogata and Yamasaki, 1997; Vincent et al., 2019). As such, these organelles supply the ATP used by myosin ATPases to achieve muscle contraction, and also play an important role in Ca^2+^ signaling (Boncompagni et al., 2009). Glancy et al. (2015) have further classified mitochondrial subpopulations based on their morphologies and proximity to various cellular structures. These uniquely situated mitochondrial structures comprise a network of interconnected organelles and serve to provide a pathway for energy distribution throughout the cell in the form of proton-motive force (PMF) (Glancy et al., 2017). Thus, the extent and location of the mitochondrial network is determined by the interplay of various ATP demand pathways, and regulated by mitochondrial quality control (MQC) machinery, which help to maintain the viability and quality of the organelle pool.

## Mitochondrial Quality Control (MQC)

**FIGURE 1:**
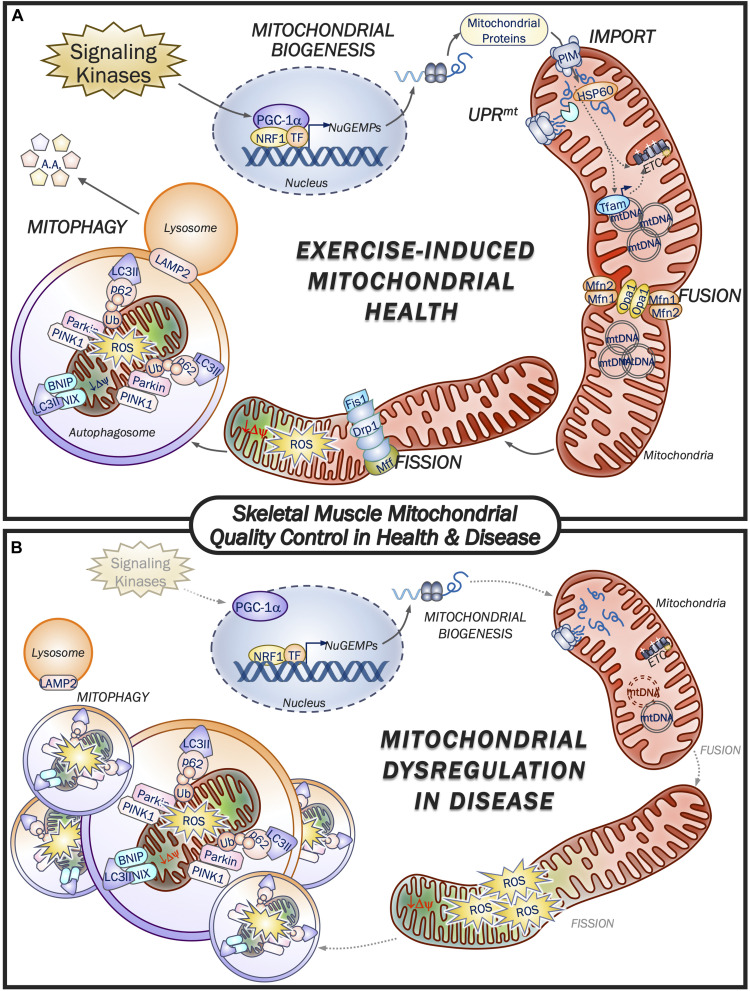
Skeletal muscle mitochondrial quality control in health and disease. **(A)** Exercise is a potent promotor of mitochondrial health by eliciting increases in mitochondrial quality control machinery. Upstream signaling kinases respond to muscle contractile activity leading to activation of PGC-1α and augmented transcription of nuclear genes encoding mitochondrial proteins (NuGEMPs). Newly translated mitochondrial-targeted proteins are then transported into the mitochondrion via the protein import machinery (PIM) and are subsequently met by resident chaperones and proteases to facilitate their incorporation within the organelle. The UPRmt is responsible for regulating the expression of mitochondrial chaperones and proteases in the presence of a stress stimulus such as exercise. As mitochondrial volume within the myofiber expands, fusion machinery adjoins neighboring organelles’ inner and outer membranes to facilitate transfer of metabolites, mtDNA, etc. Dysfunctional mitochondrial segments that lose their membrane potential and emit excessive ROS are cleaved from the reticulum by the fission proteins, thereby allowing mitophagy machinery to selectively encapsulate and degrade these non-functioning organelles for delivery to the lysosome. Exercise activates both the biogenesis and mitophagy pathways of MQC to promote the health and viability of the mitochondrial pool within muscle. **(B)** Mitochondrial dysfunction is a hallmark feature of disease, including cancer, aging and associated mitochondrial disorders. In general, activation of signaling kinases is impaired and reduces the drive for mitochondrial biogenesis from the nucleus, while mtDNA copy number can be reduced or have a high proportion of mutated mtDNA, which can serve to blunt mitochondrial expansion. Reductions in fission and fusion machinery impair mitochondrial morphology and shift the fission:fusion ratio in favor of network fragmentation and organelle ROS production. Further exacerbating the mitochondrial derangements is the impaired mitochondrial removal via reduced mitophagic clearance by the lysosome, thus contributing to the poor morphology and function of the mitochondrial network. As described in the text, exercise can reverse many of the pathways that lead to mitochondrial dysfunction, thereby restoring muscle health.

One of the most remarkable features of skeletal muscle mitochondria is the dynamic plasticity that they exhibit. For instance, prolonged aerobic exercise training is capable of eliciting improvements in mitochondrial content, mass, and overall function, whereas chronic muscle disuse produces the opposite effect (Tryon et al., 2014; Memme et al., 2019). Mitochondrial synthesis (biogenesis) is a result of increases in the expression of nuclear and mtDNA genes encoding mitochondrial proteins, while fusion proteins such as Mfn1/2 and Opa1/2 promote the merger of adjacent organelles, thus collectively expanding the mitochondrial network while improving efficiency (Iqbal et al., 2013; Mishra and Chan, 2016). Conversely, mitochondrial segments that malfunction are selectively removed, first through their segregation by fission machinery proteins such as Drp1, Fis1 and Mff in order to be cleared by the lysosome through mitophagy (Iqbal et al., 2013; Mishra and Chan, 2016). The balance of these opposing processes is imperative to mitochondrial quality and function, and any loss of equilibrium in these processes can contribute to mitochondrial impairments that are detrimental to tissue health. In fact, conditions such as advancing age, mitochondrial mutations, and diseases such as cancer are associated with deficiencies in the MQC machinery, leading to organelle dysfunction and pathogenesis (Figure 1). Conversely, exercise is perhaps the most potent stimulus for the activation of both mitochondrial biogenesis and mitophagy, and the consequent preservation and improvement of metabolic health within skeletal muscle. Therefore, it is important to understand how these processes are regulated at the molecular level.

### Mitochondrial Biogenesis and Important Signaling Proteins

Mitochondrial biogenesis is responsible for the increased synthesis of mitochondrial proteins that are derived from both nuclear and mitochondrial genomes. While there are approximately 1,200 proteins that are localized within mitochondria, mtDNA contributes a very small, but nonetheless important fraction, including 13 ETC subunit proteins synthesized by the organelle’s distinct protein synthesis machinery (Anderson et al., 1981). Signaling toward mitochondrial biogenesis is first initiated by kinases such as AMPK, p38 MAPK, CaMK, and PKA, which become activated upon the sensing of energetic and redox imbalances, increases in intracellular Ca^2+^ levels, or adrenergic signaling, respectively (Winder et al., 2000; Puigserver et al., 2001; Handschin et al., 2003; Ojuka et al., 2003; Irrcher et al., 2009; Ristow et al., 2009). These signaling kinases converge on the master regulator of mitochondrial biogenesis, PGC-1α, along with related factors such as PGC-1β and PRC, which bind various transcription factors on the promotor regions of a multitude of nuclear genes encoding mitochondrial proteins (NuGEMPs), thus increasing their expression (Hood, 2001; Handschin and Spiegelman, 2006; Scarpulla, 2011; Scarpulla et al., 2012). Perhaps the most notable PGC-1α-transcription factor interaction is through Nuclear Respiratory Factor-1 (NRF1) to promote the expression of mitochondrial transcription factor A (Tfam), which in turn mediates mtDNA transcription, thereby connecting the expression of the mitochondrial and nuclear genomes (Gordon et al., 2001; Scarpulla, 2011; Figure 1). While PGC-1α is considered to be the master regulator of mitochondrial biogenesis, it may not always be necessary to achieving mitochondrial adaptations following exercise, since training-induced increases in mitochondrial content (Leick et al., 2008) and function (Adhihetty et al., 2009; Ballmann et al., 2016) can be achieved in PGC-1 knockout animals. Indeed, additional regulators contributing to mitochondrial regulation include SirT1 (Menzies et al., 2013), which is capable of activating PGC-1α through its deacetylase function, as well as p53, another well-established transcription factor that translocates to both the nucleus and mitochondria to regulate the expression of mtDNA- and nDNA-derived transcripts (Heyne et al., 2004; Park et al., 2009; Saleem et al., 2009; Saleem and Hood, 2013; Beyfuss et al., 2018). Moreover, the regulatory regions of nuclear genes encoding mitochondrial proteins are heterogeneous and therefore, the regulation of organelle synthesis requires multiple regulators of transcription (Lenka et al., 1998; Hood, 2001). A number of transcription factors associated with exercise-induced mitochondrial biogenesis have been established, such as CREB, the estrogen related receptors (ERRα,β,**γ**), c-myc, specificity protein-1 (Sp-1), upstream stimulatory factor-1 (USF-1), as well as nuclear respiratory factors 1 and 2 (NRF1 and NRF2) (see Islam et al., 2020 for review; Hood et al., 2011; Knudsen et al., 2020). Each transcription factor when bound to target gene promoter sequences can upregulate the expression of mitochondrial proteins.

### Mitochondrial Turnover—Mitophagy and the Lysosomes

Equally important to the synthesis of new mitochondrial proteins is the removal of mitochondria that begin to emit excessive ROS, or that have lost their membrane potential, therefore becoming dysfunctional (Ljubicic et al., 2010; Lira et al., 2013; Kim et al., 2018). Mitophagy is the mitochondrial-specific form of autophagy whereby a double membrane phagophore engulfs damaged organelles to form an autophagosome, that is subsequently broken down by the lysosome and recycled (Wei et al., 2015). Although several mitophagy pathways have been elucidated, the most well-established pathway is mediated by the interaction of PINK1 and the E3-ubiquitin ligase, Parkin. Under basal conditions, PINK1 is imported into the mitochondria and degraded, however, as the mitochondrial membrane potential dissipates, PINK1 import is impaired. As a result PINK1 stabilizes on the OM and recruits Parkin, leading to the ubiquitination of OM proteins (Geisler et al., 2010). The nucleation of the phagophore membrane requires maturation of LC3-I into its lipidated form, LC3-II and is initiated by upstream activation of the Beclin1 complex. Various autophagy (ATG) proteins such as ATG7 conjugate LC3 with phosphatidylethanolamine, thus allowing for its incorporation within the membrane structure (Tanida et al., 2004). The adapter protein p62/SQSTM1 simultaneously binds to ubiquitin bound to the tagged mitochondrion along with LC3 embedded in the phagophore membrane, thus forming the autophagosome. This structure then traverses microtubule tracks to the lysosome where it can fuse, and the contents are degraded (Geisler et al., 2010; Vainshtein and Hood, 2016; Figure 1). The transcription factors TFEB and TFE3 are two critical regulators of genes involved in lysosomal biogenesis as well as autophagosomal machinery (Settembre and Ballabio, 2011; Mansueto et al., 2017). TFEB and TFE3 have been shown to be activated via common signals (i.e., AMPK, Ca^2+^) that are known to promote mitochondrial biogenesis, and they may also play a role in initiating mitochondrial biogenesis directly, or in concert with PGC-1α (Kim et al., 2014; Vainshtein and Hood, 2016; Mansueto et al., 2017; Erlich et al., 2018).

### The Mitochondrial Unfolded Protein Response (UPR^mt^)

The UPR^mt^ monitors the mitochondrial environment and appropriately mounts a response in order to either match an imposed demand, or to mitigate damage to the organelle network. In this way, the UPR^mt^ provides an intermediate between synthesis and degradation of mitochondria. Recent work has identified the transcription factor ATF4, along with its downstream targets ATF5 and CHOP, as key regulators of the UPR^mt^, which are induced by disruptions in mitochondrial proteostasis and increasing levels of ROS, such as with exercise when protein import is accelerated (Memme et al., 2016; Mesbah Moosavi and Hood, 2017; Oliveira and Hood, 2018). Together, the activation of these transcription factors provides a mitochondria-to-nucleus retrograde signal, as they translocate to the nucleus to promote the upregulation of mitochondrial chaperones and proteases to help nascent polypeptides either achieve their mature configuration, or degrade terminally misfolded proteins, respectively (Granata et al., 2017; Melber and Haynes, 2018). The proteolytic cleavage of misfolded proteins generates peptide fragments that can directly suppress organellular import, contributing to PINK1 stabilization on the outer membrane and the subsequent induction of mitophagy. Furthermore, evidence suggests that ATF4 is intricately linked to TFEB/TFE3 signaling in the presence of cellular stress (Martina et al., 2016). Thus, the UPR^mt^ matches the health status of the organelle to the appropriate signaling response and is upregulated in the early stages of an exercise stimulus, preceding improvements in mitochondrial content and function (Memme et al., 2016; Mesbah Moosavi and Hood, 2017). However, while an increasing volume of research suggests the importance of the UPR^mt^ in the maintenance of mitochondrial health, the influence of this process in disease, and the activation of the pathway by exercise remains to be fully understood.

## Mitochondrial Health With Age

The natural process of aging is associated with progressive reductions in the strength, function and size of muscle, the defining features of a condition known as sarcopenia (Rosenberg, 2011). Dysfunctional mitochondria are considered to play a primary role in the development of sarcopenia as they are important players in regulating many of the aforementioned processes that become dysregulated (Heber et al., 1996; Rapizzi et al., 2002; Kujoth et al., 2005; Schaap et al., 2006).

### Mitochondrial Content and Function During Aging

Mitochondrial decline is characteristic of aged muscle, and when viewed under the microscope, the organelle network maintains a distinctly fragmented appearance with aberrantly enlarged organelles, yet considerably thinner SS and IMF regions as compared to young muscle (Short et al., 2005; Iqbal et al., 2013; Leduc-Gaudet et al., 2015). The observed mitochondrial fragmentation can be accounted for by the reduced expression of both fission and fusion proteins, resulting in a net increase in the fission:fusion ratio (Ibebunjo et al., 2013; Iqbal et al., 2013). Diminished content is explained by reductions in both the expression of mitochondrial genes and corresponding proteins, as well as increased rates of mitophagy flux (Baraibar et al., 2013; Liu et al., 2013; Carter et al., 2015, 2018a; Chen et al., 2018). Therefore, synthesis of mitochondrial enzymes is reduced, while concurrently, mitochondrial proteins are being degraded at a faster rate. PGC-1α is likewise reduced at the transcript level in older muscle, while mtDNA mutations become more prevalent with advancing age (Wanagat et al., 2001; Bua et al., 2006; Conley et al., 2007; Chabi et al., 2008; Koltai et al., 2012; Carter et al., 2018b). The consequences of these reductions have functional implications since mitochondrial respiratory function is commonly found to be reduced, and augmented uncoupling of O_2_ consumption to the synthesis of ATP is observed (Boffoli et al., 1994; Rooyackers et al., 1996; Marcinek et al., 2005). Furthermore, as strength is correlated to muscle fiber cross sectional area, the contribution of mitochondrial derangements to muscle atrophy is considerable. Decreases in PGC-1α allow FoxO3a, a pro-atrophy gene that is suppressed by PGC-1α, to become elevated, while it has likewise been observed that atrophy is most prevalent in fibers harboring > 80% mtDNA defects (Wanagat et al., 2001; Bua et al., 2006; Sandri et al., 2006). Additionally, insufficient clearance of maladaptive organelles in aged tissue leads to overproduction of ROS, activation of apoptosis and subsequent DNA fragmentation, ultimately promoting regional atrophy along the myofiber (Bua et al., 2002; Chabi et al., 2008; Gouspillou et al., 2014).

### Exercise for the Preservation of Mitochondrial Health With Aging

The question of whether the skeletal muscle decline with aging is a byproduct of increased sedentarism or intrinsic to the aging process remains to be unequivocally determined experimentally. However, the merits of adopting a physical activity regimen is unquestionable, as continuous exercise can promote favorable organelle health, and mitigate some of the associated declines in muscle quality seen with age. Indeed, endurance training can ameliorate the decreased expression observed in many of the affected proteins that are diminished in aged muscle, such as those involved in energy metabolism and generation of ATP (Lanza et al., 2008). While life-long participation in high-level physical activity is our best means of promoting and preserving mitochondrial health in aged muscle, adoption of an exercise routine later in life is nonetheless capable of achieving levels of mitochondrial content and function that are comparable to those found in young muscle (Short et al., 2003; Lanza et al., 2008; Joseph et al., 2012; Carter et al., 2015). However, the sensitivity of aged muscle to the exercise stimulus appears to be impaired, as the activation of the important signaling kinases is blunted, as is the exercise-induced activation of mitochondrial clearance via mitophagy (Ljubicic et al., 2009; Chen et al., 2018). While basal mitophagy flux is elevated with age, it is unclear whether this is intended to be protective, or contributes to the mitochondrial defect since dysfunction still predominates aged muscle, as evidenced by increased ROS emission (O’Leary et al., 2013; Carter et al., 2018a; Chen et al., 2018). Nevertheless, exercise can mitigate the excessive accumulation of ROS and attenuate activation of atrophy-inducing apoptosis (Ljubicic et al., 2009; Di Meo et al., 2019). Exercise also reverses the age-induced reductions in PGC-1αtranscription, restoring it to levels observed in younger cohorts (Carter et al., 2018b). In addition to endurance exercise, resistance type training also promotes muscle health by promoting muscle hypertrophy while also recruiting the fusion of satellite cells that do not harbor mtDNA mutations, despite the reduced stimulus for mitochondrial biogenesis that this training modality offers (Taivassalo, 1999; Kowald and Kirkwood, 2014; Carter et al., 2015). Thus, continuous participation in regular exercise training, whether adopted throughout life, or much later, is an established and non-pharmacological approach to maintaining mitochondrial health, leading to improved skeletal muscle function and overall quality of life.

## Muscle-Specific Mitochondrial Mutations and Mitochondrial Disorders

As mitochondria are integral organelles in the maintenance of many cellular processes, the pathogenesis of various diseases can be traced to mitochondrial irregularities that may be inherent or acquired via lifestyle factors. More than 300 disease-associated mtDNA mutations have been identified, which can be attributed to either genetic inheritance, or the vulnerability of mtDNA and its exposure to high levels of ROS in the mitochondrial matrix (Li et al., 2019). However, the investigation of mitochondrial disorders (MD) is complex, and often present with varied pathological phenotypes that manifest in a range of tissues including brain, liver, eye, heart, and skeletal muscle (Alston et al., 2017).

### Mutations and Manifestation of Mitochondrial Disorders

Mutations to either mitochondrial or nuclear-derived genes can produce mitochondrial defects that most often present within skeletal muscle, either as the lone affected tissue or as a predominant feature of a multisystem disorder, thus making it the tissue of choice for the study of MD (Taylor et al., 2004; McFarland et al., 2010). Skeletal muscle can be routinely sampled via the muscle biopsy technique, which allows full characterization of the functional and morphological impairments to mitochondria (Phadke, 2017). Mutations of mtDNA commonly affect mitochondrial tRNAs and protein encoding regions, and are associated with mtDNA instability leading to derangements in copy number and quality (Russell and Turnbull, 2014; Rusecka et al., 2018). The broad range of mutations can impact various aspects of mitochondrial quality control, not limited to an effect on ETC function or impaired ATP production. Clinical detection of MD can be made histologically via the presence of ragged red fibers (RRFs), COX-negative fibers, as well as SDH deficiency (Phadke, 2017). Among the numerous MDs, common diseases include MELAS, Kearns-Sayre syndrome, Leigh syndrome, MERRF, and rhabdomyolysis, which present with weakness, fatigue and severe myopathy among a spectrum of other symptoms (Taylor and Turnbull, 2005; Alston et al., 2017; Phadke, 2017; Rusecka et al., 2018; Yan et al., 2019). Additionally, derangements in nuclear-transcribed mitochondrial genes can contribute to the etiology of various other conditions (Li et al., 2019; Sharma and Sampath, 2019). For instance, mutations in the Tafazzin gene promotes skeletal muscle and cardiac myopathy associated with Barth Syndrome due to impaired maturation of the inner membrane phospholipid cardiolipin that is required for membrane stability (Schlame and Ren, 2006). Likewise, defects in the mitophagy protein parkin prevents the clearance of dysfunctional mitochondria, contributing to the development of Parkinson’s disease (Dawson and Dawson, 2010). Evidently, the multifaceted regulation of mitochondrial content, morphology and function presents numerous opportunities for mutation and the development of various diseases.

### Exercise as Therapy for Mitochondrial Disorders

The reliance of mitochondria on nuclear-encoded genes that are essential for mtDNA transcription makes the development of targeted therapies difficult. However, exercise is a proven stimulus for the improvement of mitochondrial content and function within skeletal muscle, and despite a reduced exercise capacity in patients with MD, evidence suggests that adhering to a continuous exercise regimen may serve as the most complete therapeutic intervention for the restoration of mitochondrial function. Preclinical studies have shown that PGC-1α may provide a useful therapeutic target as its activation may alleviate some of the observed myopathy and mitochondrial respiratory defects that are present (Srivastava et al., 2009; Fiuza-Luces et al., 2019). As such, the ability of endurance exercise to activate multiple signaling kinases that converge on PGC-1α suggests that it is a potent stimulus. Studies have illustrated that exercise is a viable intervention strategy that is effective in increasing citrate synthase activity, ETC complexes and overall patient aerobic fitness (Taivassalo et al., 1999, 2001, 2006; Jeppesen et al., 2006, 2009; Porcelli et al., 2016; Fiuza-Luces et al., 2018). Similar to the benefits described in aged muscle, strength training may also aid in promoting muscle mass and strength in MD patients, while reducing mtDNA heteroplasmy as a result of satellite cell recruitment, without any observable negative secondary effects (Kraemer et al., 2002; Murphy et al., 2008; Groennebaek and Vissing, 2017). Moreover, the combination of resistance with endurance training promoted significant improvements in the quality and function of skeletal and respiratory muscle in MD patients (Fiuza-Luces et al., 2018). Studies employing the mtDNA mutator mice, which harbor mutations in mitochondrial polymerase γ, have also suggested a potential role for exercise-induced p53 localization to the mitochondria to aid in the restoration of organellular morphological and functional defects (Safdar et al., 2016). Interestingly, there is evidence to suggest that endurance training promotes the restoration of mitochondrial defects associated with mtDNA mutations in the brains of patients with neurogenerative diseases such as Alzheimer’s (Picard et al., 2016). Thus, exercise elicits multiple signaling cascades that improve mitochondrial health, and does so across a range of affected tissues, which is particularly important given the multifaceted nature of MD.

## Cancer Cachexia

While mitochondrial dysfunction is a hallmark feature of cancer tumorigenesis, in skeletal muscle, mitochondrial dysfunction contributes to the progressive muscle wasting associated with cancer cachexia. Approximately 80% of late stage cancer patients experience cachexia, which impacts morbidity and contributes to 20–40% of all cancer mortalities (Melstrom et al., 2007; Tisdale, 2009; Prado et al., 2013). The consensus definition of cachexia describes three stages of the condition: (1) Precachexia, involving weight loss < 5% along with signs of anorexia and impaired glucose tolerance; (2) cachexia, diagnosed when weight loss exceeds 2–5%; and (3) refractory cachexia, referring to variable levels of muscle wasting whereby the cancer is highly pro-catabolic with poor prognosis (Fearon et al., 2011). Thus, treatments are most effective when administered early, however, clinical management remains a major challenge as patient population, stage, type of tumor, and chemotherapies may contribute to the progression of cachexia.

### Signaling for Cancer Cachexia and the Role of Mitochondria

The prevailing belief is that the progression of cachexia is the result of tumor-induced immune cell activation leading to systemic inflammation and the release of potent cytokines TNFα, IFNγ, IL-1, and IL-6, which promote changes in metabolism, energy wasting, cancer-induced anorexia, and skeletal muscle protein catabolism (Dwarkasing et al., 2016; Van Norren et al., 2017). The progressive nature of cachexia is based on the convergence of imbalanced proteostasis, myofiber degeneration and structural remodeling, as well as altered mitochondrial function (Van Der Ende et al., 2018). Overactivation of the ubiquitin proteasome system (UPS) and the autophagy-lysosome system contribute to advanced protein turnover, while impaired mTOR signaling contributes to impaired synthesis of new proteins (Llovera et al., 1995; Lorite et al., 1998; Gordon et al., 2013; Sandri, 2016). A consistent feature of cancer cachexia is the vast remodeling of the mitochondrial network and the array of changes in the expression level of regulatory genes and proteins involved in mitochondrial biogenesis (Cornwell et al., 2014; Judge et al., 2014; Shum et al., 2015). Most studies indicate that PGC-1α protein is significantly reduced with cancer cachexia, while genome-wide transcriptome datasets show that PGC-1β gene expression is similarly diminished (Fontes-Oliveira et al., 2013; Puppa et al., 2014; Sheng et al., 2017; Van Der Ende et al., 2018). Activators of PGC-1α SirT1 and MEF2C, are also found to decrease in cachectic muscle, as well as downstream targets, ERRα and Tfam, altogether indicating impaired drive for organelle synthesis (White et al., 2011; Shum et al., 2012; Zhuang et al., 2016; Chen et al., 2017; Van Der Ende et al., 2018). Indeed, it appears that the suppression of PGC-1α is a key event in the progression of cancer cachexia and is directly related to elevations in circulating IL-6 (Baltgalvis et al., 2008; White et al., 2012). Additionally, morphology proteins, Drp1, along with Opa1 and Mfn2 are reduced in cancer- and chemotherapy-induced cachexia, thus leading to increased organelle toxicity, and reduced organelle efficiency (Barreto et al., 2016; Van Der Ende et al., 2018). Microscopically, mitochondria in cachectic tissue appear swollen and are often contained in vesicle-like structures indicative of their incomplete clearance by the mitophagy machinery (Fontes-Oliveira et al., 2013; Tzika et al., 2013; Pin et al., 2015). Coincidentally, increases in autophagosomal markers such as p62, BNIP3, and the LC3-II:LC3-I ratio, along with the appearance of undegraded mitochondria further suggest impairments in the function of the lysosomes and their ability to fuse with, and digest the fully formed mitochondria-containing autophagosomes (Pin et al., 2015; Brown et al., 2017; Segatto et al., 2017).

Although more work is needed in this area, an increasing volume of evidence suggests that impairments in mitochondrial function play a causal role in the muscle wasting observed in cancer. In particular, over a 4 weeks time course following C26 tumor cell inoculation, one study found that changes in mitochondrial content and function preceded the corresponding atrophy, while another reported that mitochondrial dysfunction directly influenced amino acid metabolism, and diminished protein synthesis (Brown et al., 2017; Kunzke et al., 2020). Interestingly, a study in which Drp1 was selectively knocked out within rodent muscle demonstrated the robust myopathy, fiber degeneration/regeneration, and mitochondrial impairments that are characteristic of cachexia (Favaro et al., 2019). Notably, the corresponding mitochondrial dysfunction in these animals lead to the activation of ATF4, consequently inducing release of the myokine FGF21, which largely accounted for the diminished body mass (Favaro et al., 2019). These reports highlight a primary role for mitochondria in the pathogenesis of cancer cachexia and suggest the importance of targeted therapies that improve the mitochondrial condition, thus helping to suppress muscle wasting.

### Exercise as Mitochondrial Medicine for Cancer Cachexia

It is clear that mitochondria are highly relevant to not only tumorigenesis but also the progression of cancer cachexia. Therefore, it is reasonable to presume that regular exercise, with its well known effect on improving mitochondrial content and function, would provide a potent and viable therapeutic option for cancer patients. Multiple studies in both rodent and human tissue have reported that continuous exercise training in cachectic patients is capable of improving muscle mass and strength (Deuster et al., 1985; Salomão et al., 2010; Penna et al., 2011). Interestingly, both endurance and resistance type training modalities are sufficient to elicit these responses (Norton et al., 1979; Jaweed et al., 1983; Otis et al., 2007; Schmidt et al., 2015; Ballarò et al., 2019a; Sato et al., 2019). While considerable work is still required to dissect the molecular mechanisms at play, the current literature suggest that the signaling events associated with exercise reverse the pro-cachectic signals that promote skeletal muscle decline and frailty. Treadmill training has been also been shown to similarly augment mTOR signaling and augment mitochondrial quality control in the presence of systemic IL-6 overexpression, a major contributor to the activation of cachexia in cancer patients (White et al., 2012, 2013). Along with activation of PGC-1α, exercise also signals improvements in mitochondrial quality by mediating the expression of mitophagy proteins such as BNIP3, PINK1, and Parkin, along with fission/fusion machinery, and reduces ROS via augmented redox homeostasis (Pigna et al., 2016; Ballarò et al., 2019a,b). Moreover, the exercise stimulus is capable of enhancing oxidative capacity in tumor bearing mice, and aerobic fitness was found to be directly correlated to survival, suggesting the importance of mitochondrial health in tumor-bearing subjects (Hardee et al., 2016; Pigna et al., 2016). In addition, patients diagnosed with p53-mutation cancers, such as soft-tissue sarcomas, or cancers of the colon, lung, breast, liver, brain, and hemopoietic tissues, are likely able to improve muscle health through exercise training. p53 maintains normal mitochondrial content and function in muscle, but when mutated, leads to reduced mitochondrial function and causes cancer. Studies initially by Saleem et al. (2009), and subsequently by Beyfuss et al. (2018) found that muscle mitochondrial content and function can still adapt to exercise in the absence of p53, and muscle health benefits are still possible. However, it should also be noted that in animals studies involving late stage cachexia, exercise had no beneficial effect, and in fact reduced their survival. Thus, it appears that exercise is most effective as an early intervention strategy to either prevent or to slow down cachexia progression. The optimal exercise parameters, such as type, duration, intensity and frequency, to properly prescribe as part of training program to patients remains to be determined. However, it is clear that exercise provides a multifactorial treatment option that can be employed early in a patient’s diagnosis to improve strength, mobility, energy metabolism and overall quality of life.

## Summary and Future Perspectives

As mitochondrial quality control is highly dynamic and relies on the convergence of multiple processes to regulate the function of the organelle network, the development of therapeutic interventions that adequately address all aspects of mitochondrial remodeling remains a challenge to identify. Moreover, the metabolic nature of skeletal muscle, and the relative proportion of body mass that it comprises, make muscle a unique tissue to study in the context of various diseases. Exercise performed on a regular basis in either continuous or interval training formats remains our one true treatment modality capable of augmenting mitochondrial health in muscle, by coordinately inducing both synthesis and recycling of mitochondrial proteins, and therefore serving as mitochondrial medicine for muscle. Indeed, this signaling toward mitochondrial turnover begins with the very first bout of exercise, which triggers the onset of biogenesis as well as mitophagy (Vainshtein and Hood, 2016). Ultimately, long-term training-induced adaptations can also be used to identify specific factors that can potentially be targeted pharmaceutically as adjuncts to promote mitochondrial health in the presence of an exercise stimulus, or perhaps independently for those incapable of adopting a training program. However, before this can be accomplished, there are still many aspects of mitochondrial signaling associated with exercise that remain elusive. For instance, while the balance of synthesis and degradation is well understood, other key regulators of mitochondrial remodeling require further analyses, such as the UPR^mt^, as well as TFEB and TFE3 as master regulators of lysosomal biogenesis and autophagy proteins. The interplay of these processes and their regulators with exercise warrants considerable research effort to establish a clear understanding of the mechanistic features that drive the sustenance of a high quality mitochondrial pool, and the consequent maintenance of muscle health with age, cancer and mitochondrial disease.

## Author Contributions

JM and DH contributed to writing, editing and reviewing this manuscript, and accompanying figure. Both authors contributed to the article and approved the submitted version.

## Conflict of Interest

The authors declare that the research was conducted in the absence of any commercial or financial relationships that could be construed as a potential conflict of interest.

## Abbreviations

AMP: adenosine monophosphate
AMPK: AMP-activated protein kinase
ATF4: activating transcription factor 4
ATF5: activating transcription factor 5
ATP: adenosine triphosphate
BNIP3: BCL2/adenovirus E1B 19 kDa protein-interacting protein 3
C26: colon-26 adenocarcinoma tumor cells
CaMK: Ca2+/calmodulin-dependent protein kinase
CHOP: CCAAT/enhancer-binding protein (C/EBP) homologous protein
COX: cytochrome c oxidase
Drp1: dynamin related protein 1
eIF2 α: eukaryotic initiation factor 2 α
ERR α: estrogen related receptor α
ETC: electron transport chain
FGF21: fibroblast growth factor 21
Fis1: mitochondrial fission protein 1
FoxO3a: forkhead box O3a
IFN γ: interferon γ
IL-1: interleukin 1
IL-6: interleukin 6
IM: inner mitochondrial membrane
IMF: intramyofibrillar
LC3-I/II: microtubule-associated proteins 1A/1B light chain 3
MD: mitochondrial disorder
MEF2C: myocyte enhancer factor 2C
MELAS, mitochondrial myopathy: encephalomyopathy, lactic acidosis and stroke-like episodes
Mff: mitochondrial fission factor
Mfn1/2: mitofusin 1&2
MQC: mitochondrial quality control
mtDNA: mitochondrial DNA
mTOR: mammalian target of rapamycin
NIX: BNIP3-like protein
NRF-1: nuclear respiratory factor 1
OM: outer mitochondrial membrane
Opa1/2: optic atrophy 1&2
p38 MAPK: p38 mitogen activated protein kinase
p53: tumor protein 53
p62: ubiquitin-binding protein p62 aka sequestosome 1
PIM: protein import machinery
PINK1: PTEN-induced kinase 1
PKA: protein kinase A
PRC: PGC-related co-activator
ROS: reactive oxygen species
PGC-1 α/β: PPAR γ coactivator, 1 α &1 β
RRF: ragged red fibers
SDH: succinate dehydrogenase
Sirt1: sirtuin 1
SS: subsarcolemmal
Tfam: mitochondrial transcription factor A
TFE3: transcription factor binding to IGHM enhancer 3
TFEB: transcription factor EB
TNF α: tumor necrosis factor α
UPR^mt^: mitochondrial unfolded protein response
UPS: ubiquitin proteosome system
VDAC: voltage dependent anion channel.
